# Therapeutic Potential of Momordicine I from *Momordica charantia*: Cardiovascular Benefits and Mechanisms

**DOI:** 10.3390/ijms251910518

**Published:** 2024-09-29

**Authors:** Pai-Feng Kao, Chun-Han Cheng, Tzu-Hurng Cheng, Ju-Chi Liu, Li-Chin Sung

**Affiliations:** 1 Division of Cardiology, Department of Internal Medicine, Shuang Ho Hospital, Ministry of Health and Welfare, Taipei Medical University, New Taipei City 23561, Taiwan; 12318@s.tmu.edu.tw (P.-F.K.); liumdcv@s.tmu.edu.tw (J.-C.L.); 2 Department of Medical Education, Linkou Chang Gung Memorial Hospital, Taoyuan City 33305, Taiwan; leocheng1991@gmail.com; 3 Department of Biochemistry, School of Medicine, College of Medicine, China Medical University, Taichung City 404333, Taiwan; thcheng@mail.cmu.edu.tw; 4 Division of Cardiology, Department of Internal Medicine, School of Medicine, College of Medicine, Taipei Medical University, Taipei 11002, Taiwan; 5 Taipei Heart Institute, Taipei Medical University, Taipei 11002, Taiwan

**Keywords:** *Momordica charantia*, momordicine I, cardiovascular benefits, inflammation, pharmacokinetics

## Abstract

*Momordica charantia* (bitter melon), a traditional medicinal plant, has been demonstrated to have potential in managing diabetes, gastrointestinal problems, and infections. Among its bioactive compounds, momordicine I, a cucurbitane-type triterpenoid, has attracted attention due to its substantial biological activities. Preclinical studies have indicated that momordicine I possesses antihypertensive, anti-inflammatory, antihypertrophic, antifibrotic, and antioxidative properties, indicating its potential as a therapeutic agent for cardiovascular diseases. Its mechanisms of action include modulating insulin signaling, inhibiting inflammatory pathways, and inducing apoptosis in cancer cells. The proposed mechanistic pathways through which momordicine I exerts its cardiovascular benefits are via the modulation of nitric oxide, angiotensin-converting enzymes, phosphoinositide 3-kinase (PI3K)/ protein kinase B (Akt), oxidative stress, apoptosis and inflammatory pathways. Furthermore, the anti-inflammatory effects of momordicine I are pivotal. Momordicine I might reduce inflammation through the following mechanisms: inhibiting pro-inflammatory cytokines, reducing adhesion molecules expression, suppressing NF-κB activation, modulating the Nrf2 pathway and suppressing c-Met/STAT3 pathway. However, its therapeutic use requires the careful consideration of potential side effects, contraindications, and drug interactions. Future research should focus on elucidating the precise mechanisms of momordicine I, validating its efficacy and safety through clinical trials, and exploring its pharmacokinetics. If proven effective, momordicine I could considerably affect clinical cardiology by acting as a novel adjunct or alternative therapy for cardiovascular diseases. To date, no review article has been published on the role of bitter-melon bioactive metabolites in cardiovascular prevention and therapy. The present work constitutes a comprehensive, up-to-date review of the literature, which highlights the promising therapeutic potential of momordicine I on the cardiovascular system and discusses future research recommendations.

## 1. Introduction

### 1.1. Background on Momordica charantia (Bitter Melon)

**Figure 1:**
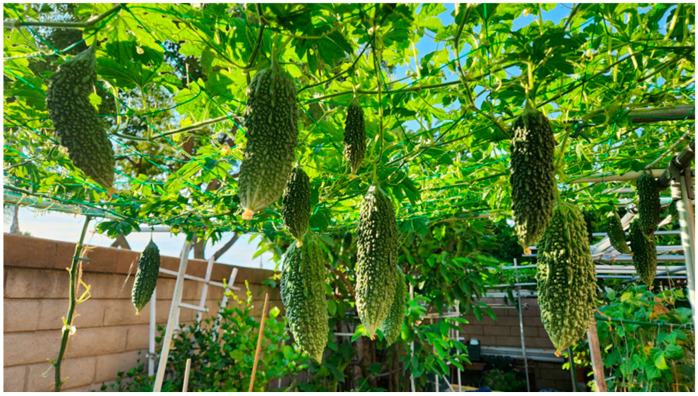
The picture shows the morphological characteristics of the *Momordica charantia* (bitter melon) fruits.

*Momordica charantia*, commonly known as bitter melon (Figure 1), is a tropical vine belonging to the Cucurbitaceae family and is cultivated in Asia, Africa, and the Caribbean. Bitter melon has been traditionally used in Ayurveda and Traditional Chinese Medicine for managing diabetes, gastrointestinal conditions (e.g., diarrhea and colic), and infections. The characteristic bitter taste of bitter melon aids in digestion and detoxification. Recent studies have identified various bioactive compounds in bitter melon, including saponins, sterols, polysaccharides, triterpenes, alkaloids, and phenolic compounds, that exhibit antioxidant, anticancer, anti-obesity, anti-inflammatory, and antidiabetic properties [1,2,3]. These findings highlight the therapeutic potential of bitter melon and indicate how its traditional uses and bioactive components are relevant and beneficial in modern healthcare applications [4].

### 1.2. Introduction to Momordicine I

**Figure 2:**
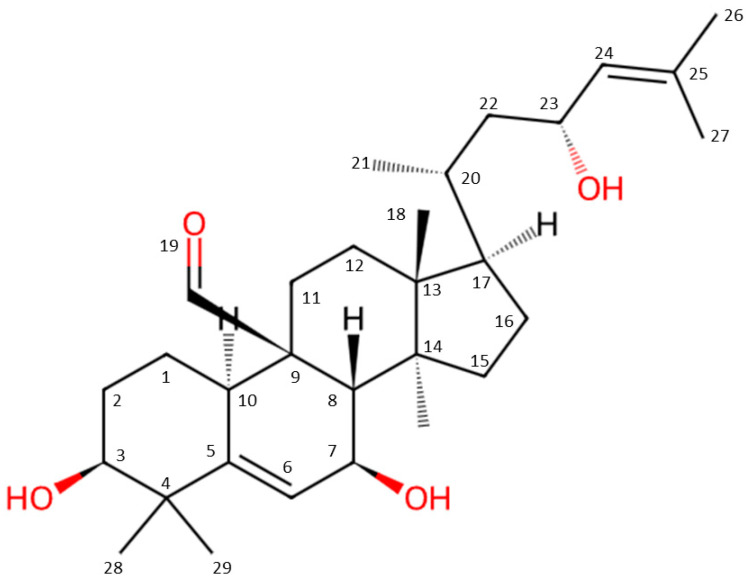
Chemical structure and atomic numbering of momordicine I. This content was adapted from ChemSpider chemistry database [6].

Momordicine I, a cucurbitane-type triterpenoid isolated from *M. charantia*, was first identified in 1984 (Figure 2) [5,6]. This bioactive compound has attracted considerable attention because of its potent biological activities. Momordicine I is a cucurbitane-type triterpenoid found in the vines and leaves of bitter melon and contributes to its therapeutic potential. A study reported that momordicine I exhibits strong antihyperglycemic activity and is thus a promising candidate for diabetes management [7]. Moreover, momordicine I was found to inhibit high-glucose-induced proliferation and collagen synthesis in rat cardiac fibroblasts, suggesting its protective effect against cardiac complications associated with diabetes [8]. Furthermore, studies examining the effect of momordicine I on various cancer cell lines and inflammation models have reported that it exerts cytotoxic and anti-inflammatory effects [9]. A recent study demonstrated that momordicine I could suppress the growth of head and neck cancer by altering the immunosuppressive effects of tumor-infiltrating macrophages and B lymphocytes [10]. These diverse properties indicate the potential of momordicine I as a valuable therapeutic agent in modern medicine [11]. The aim of this review is to investigate the role of momordicine I as a natural compound in cardiovascular prevention and therapy. In this work, the potential molecular pathway and comprehensive evidence of momordicine I’s beneficial effects on the cardiovascular system will be thoroughly discussed.

## 2. Chemical Properties and Mechanism of Action

### 2.1. Chemical Structure and Properties

The methanol extract of *M. charantia* contains various cucurbitane-type triterpenoids, which are mainly responsible for its pharmacological effects [11]. Among these, momordicine I and II are particularly known for their bioactivity. They have only slight structural differences [12]. Momordicine I can be extracted from the stems, leaves and fruits of *M. charantia*. Momordicine II can be isolated from the leaves of *M. charantia* [13]. In previous a report, momordicine I, not momordicine II, possessed an anti-inflammatory effect via the inhibition of inducible nitric oxide synthase (iNOS) in lipopolysaccharide (LPS)-treated RAW 264.7 cells [9]. Momordicine I [C30H48O4, ChemSpider ID: 95601787; IUPAC Name: 3,7-dihydroxy-17-(4-hydroxy-6-methylhept-5-en-2-yl)-4,4,13,14-tetramethyl-2,3,7,8,10,11,12,15,16,17-decahydro-1H-cyclopenta[a]phenanthrene-9-carbaldehyde] with the molecular weight of 472.710 g/mol is a white crystalline solid [6,14]. Cucurbitane-type triterpenoids possess a tetracyclic triterpene backbone with various functional groups attached, which enhance their biological activity [11,15,16]. The structure of these compounds includes a cucurbitane skeleton with multiple hydroxyl groups and a carboxyl group, which are critical for their interaction with biological targets. These characteristics contribute to the substantial cytotoxic and anti-inflammatory properties of these compounds, likely through the modulation of key signaling pathways involved in cell proliferation and inflammation [9]. Studies have investigated the transport properties of these triterpenoids and demonstrated that they can effectively cross the human intestinal epithelial cell Caco-2 monolayer—an in vitro model for intestinal absorption. Previous studies have determined that cucurbitane-type triterpenoids are not altered during their metabolism [15,17]. This finding suggests that cucurbitane-type triterpenoids maintain high bioavailability when consumed orally, which is crucial for their potential use as therapeutic agents [15]. Moreover, the stems and leaves of *M. charantia* contain other cucurbitane-type triterpenoids, such as 3β,7β,25-trihydroxycucurbita-5,23(E)-dien-19-al and 3β,25-dihydroxycucurbita-5,23(E)-dien-19-al. These compounds exhibit diverse pharmacological activities, including antidiabetic and anticancer effects [5,16]. The unique chemical structures of these triterpenoids, characterized by specific functional groups, can help us understand their mechanisms of action and potential therapeutic applications.

### 2.2. Pharmacodynamics and Pharmacokinetics

Momordicine I exhibits crucial pharmacodynamic and pharmacokinetic properties. Pharmacodynamically, momordicine I demonstrates potent antidiabetic, anti-inflammatory, and anticancer activities. Momordicine I exerts an antidiabetic effect by modulating insulin signaling pathways, enhancing glucose uptake, and inhibiting glucose production in the liver [18]. Previous studies have reported that the methanol extract of *M. charantia* exerted hepato-renal protective effects on streptozotocin-induced diabetic male rats and an antihypertensive effect on Dahl salt-sensitive rats with high-salt-induced hypertension [19,20]. Momordicine I exerts an anti-inflammatory effect by downregulating proinflammatory cytokines and inhibiting the nuclear factor kappa-light-chain enhancer of the activated B-cell (NF-κB) signaling pathway [9]. Momordicine I exerts anticancer effects by suppressing tumor growth, inhibiting glucose and lipid metabolism, enhancing the immune defense system, and inducing apoptosis in cancer cells. In particular, momordicine I targets the c-mesenchymal–epithelial transition factor (c-Met) and disrupts downstream signaling pathways by inactivating the signal transducer and activator of transcription 3 (STAT3) [14]. A study reported that momordicine I was nontoxic and remained stable in the blood of male C57Bl/6 mice. As aforementioned, in an LPS-induced RAW 264.7 cell model, treatment with 1–10 µM momordicine I inhibited NF-κB-mediated iNOS expression in a dose-dependent manner, demonstrating its anti-inflammatory activity [9]. 

Pharmacokinetic studies on the absorption, distribution, metabolism, and excretion (ADME) of momordicine I have indicated that it is efficiently absorbed in the gastrointestinal tract. Wu et al. (2014) determined that momordicine I can effectively cross Caco-2 cell monolayers, indicating its high intestinal absorption [15]. Once absorbed, momordicine I is distributed to various tissues, including the liver, where it undergoes extensive metabolism. The resulting metabolites are primarily excreted through the bile, and a minor portion is also eliminated through the kidneys. Advanced bioinformatics and computational modeling studies have provided deeper insights into the binding interactions and stability of momordicine I in biological systems. These studies have demonstrated that momordicine I has a high binding affinity for key protein targets in metabolic and inflammatory pathways and thus has therapeutic potential [21]. Moreover, molecular docking studies have reported that momordicine I can noncovalently inhibit critical enzymes, such as SARS-CoV-2 protease, indicating its potential role in antiviral therapy [22]. Overall, studies on the pharmacodynamics and pharmacokinetics of momordicine I have demonstrated its efficacy and potential as a therapeutic agent, with broad implications for managing diabetes, inflammation-associated diseases, and cancer.

### 2.3. Mechanisms of Action

Momordicine I exhibits diverse biological activities through several mechanisms of action. It primarily exerts its effects by modulating key signaling pathways involved in glucose metabolism, inflammation, and cancer. A major mechanism of action of momordicine I is its antihyperglycemic effect. This compound enhances insulin secretion and improves glucose uptake in peripheral tissues. In particular, momordicine I activates the AMP-activated protein kinase (AMPK) pathway, which is crucial for maintaining energy homeostasis. This activation stimulates the translocation of glucose transporter type 4 to the cell membrane, promoting glucose uptake and fatty acid oxidation while simultaneously inhibiting gluconeogenesis in the liver [7,23]. In addition, momordicine I and other triterpenoid components of *M. charantia* modulate the gut microbiota and increase the production of short-chain fatty acids (SCFAs), thereby contributing to its lipid-lowering effect in hyperlipidemic mice [24]. In terms of anti-inflammatory action, momordicine I and its isomer inhibit the TLR4/MyD88/kappa B kinase/NF-κB signaling pathway, thereby reducing the expression of proinflammatory cytokines, such as iNOS, tumor necrosis factor-α (TNF-α), and interleukin-6 (IL-6) [9]. Furthermore, momordicine I and its isomer enhance the expression of nuclear factor erythroid 2-related factor 2/heme oxygenase-1 (Nrf2/HO-1) by activating mitogen-activated protein kinases (MAPKs), including p38 and extracellular signal-related kinase 1/2. Nrf2 is a basic leucine zipper transcription factor that regulates the expression of several antioxidant enzymes in cells. Normally, Nrf2 is kept in the cytoplasm. However, under excessive oxidative stress, Nrf2 is translocated into the nucleus, where it activates the antioxidant genes to maintain redox homeostasis and cell survival. The excessive oxidative stress is characterized by elevated intracellular levels of reactive oxygen species (ROS), which play a key role in inducing inflammation [25,26]. This anti-inflammatory effect is crucial for preventing chronic inflammation–related diseases, such as cardiovascular disease, diabetes and cancer progression. Furthermore, momordicine I exhibits substantial anticancer properties: it induces apoptosis in cancer cells by activating caspases and promoting the release of cytochrome c from mitochondria. In addition, momordicine I inhibits cancer cell proliferation by downregulating the c-Met/STAT3 signaling pathway, which plays a critical role in cell growth and survival [14]. In glioma cells, momordicine I disrupts mitochondrial oxidative phosphorylation, leading to decreased ATP production and increased cell death [27]. Moreover, momordicine I (at concentrations of 0.1–1 µM) exerts a cardioprotective effect by suppressing high-glucose-induced ROS production; it achieves this effect by activating the antioxidant Nrf2/HO-1 pathway and inhibiting the transforming growth factor-*β1* (TGF-β1) suppressor of mothers against the decapentaplegic 2/3 signaling pathway. Furthermore, momordicine I reduces high-glucose-induced proliferation and collagen synthesis in cardiac fibroblasts, which helps prevent cardiac fibrosis and related complications in patients with diabetes [8]. In addition, treatment with 12.5 µM momordicine I alleviated isoproterenol-induced cardiomyocyte hypertrophy by suppressing the expression of phospholipase A2 group VI (PLA2G6) and diacylglycerol kinase-ζ (DGK-ζ), which are key enzymes crucial for lipid signaling and inflammation [28]. In conclusion, the diverse mechanisms of action of momordicine I indicate its potential as a therapeutic agent for managing diabetes, inflammation, cancer, and cardiovascular diseases. Moreover, the ability of momordicine I to modulate key signaling pathways and metabolic processes highlights its importance in both traditional and modern medicinal contexts.

## 3. Cardiovascular Effects of Momordicine I

### 3.1. Preclinical Studies

Preclinical studies have provided substantial evidence of the cardiovascular benefits of momordicine I, highlighting its potential in managing and preventing cardiovascular diseases through various mechanisms. Specifically, momordicine I can mitigate cardiac hypertrophy, a condition characterized by the abnormal enlargement of heart muscle cells that can lead to cardiomyopathy and heart failure. Li et al. (2023) demonstrated that momordicine I alleviated isoproterenol-induced cardiomyocyte hypertrophy in rats by suppressing the expression of PLA2G6 and DGK-ζ enzymes involved in the glycerophospholipid metabolic pathway [28]. This suppression reduced lipid accumulation and inflammation, thereby protecting cardiac cells from hypertrophic damage [28]. In addition to its effects on cardiac hypertrophy, momordicine I could effectively reduce high-glucose-induced proliferation and collagen synthesis in rat cardiac fibroblasts. This activity is crucial for preventing cardiac fibrosis, a condition that leads to the stiffening and malfunctioning of the heart muscle [8]. Chen et al. (2018) determined that momordicine I inhibits cardiac fibroblast proliferation and reduces collagen production, thus protecting the heart from fibrotic changes that are common in diabetes [8]. Moreover, the antioxidative properties of momordicine I considerably enhance its cardiovascular protective effects. Momordicine I reduces ROS, a key factor in the development of cardiovascular diseases [29]. By inhibiting ROS production and enhancing antioxidant enzyme activity, momordicine I maintains the integrity and function of endothelial cells, which line the blood vessels and play a crucial role in vascular health [27,29]. The potential anti-inflammatory effects of momordicine I play a crucial role in cardiovascular protection. Momordicine I inhibits the activation of the NF-κB pathway, a major regulator of inflammation. This inhibition leads to a reduction in the expression of proinflammatory cytokines, such as TNF-α and IL-6, thus preventing chronic inflammation that can damage the cardiovascular system [9]. Chang et al. have identified the bioactive components of *M. charantia* that act as glucagon-like peptide 1 (GLP-1) secretagogues, offering further cardiovascular benefits by affecting enteroendocrine cells [30,31]. Furthermore, computational studies have supported these findings by identifying the molecular interactions and binding affinities of momordicine I with various cardiovascular-related targets. For instance, a docking study suggested that momordicine I effectively binds to enzymes involved in lipid metabolism and inflammatory pathways, indicating its therapeutic potential in cardiovascular diseases [32]. For example, alterations in glycerophospholipid metabolism were identified as predictors of coronary artery disease (CAD) progression in clinical observations [33]. Overall, preclinical studies have demonstrated the potential of momordicine I as a diverse therapeutic agent in cardiovascular health, highlighting its ability to combat hypertrophy, fibrosis, oxidative stress, and inflammation. Conventional medical therapies are associated with high costs, limited efficacy, and substantial side effects. Given these promising findings, further investigation in clinical settings is warranted to confirm the efficacy and safety of momordicine I in human populations.

### 3.2. Mechanisms and Potential Therapeutic Applications

Numerous studies have confirmed the nutraceutical properties of bitter melon (Table 1). Multiple mechanisms of action are responsible for the beneficial cardiovascular effects of *M. charantia* and its bioactive compounds, including their antioxidative, antihypertensive, antifibrosis, antihypertrophic, lipid-lowering, anticancer, anti-inflammatory, and antidiabetic properties. 

Diabetes mellitus frequently results in cardiovascular complications due to metabolic dysregulation. *M. charantia*, which is particularly rich in momordicine I, has been widely studied for its antidiabetic effects (Table 1). Cucurbitane-type triterpene glycosides from *M. charantia* were observed to exert an inhibitory effect on α-amylase and α-glucosidase both in vitro and in vivo [35]. Studies have indicated that *M. charantia* can enhance insulin sensitivity through various mechanisms. Triterpenoids in *M. charantia* stimulate GLUT-4 translocation, activate the AMPK signaling pathway, and increase PPAR-γ expression in the white adipose tissue [7,23]. A similar effect was observed in C57BL/6J mice on a high-fat diet, where *M. charantia* fruit extract increased the mRNA expression of PPAR-γ in the adipose tissue [53]. Furthermore, the bioactive components of *M. charantia* act as GLP-1 secretagogues [30,31]. Although all clinical human studies on *M. charantia* have demonstrated hypoglycemic effects, results concerning the reduction in glycated hemoglobin levels have been inconsistent [46,49,50,51,54]. Bitter melon also exhibits strong antioxidant properties. Various in vitro and in vivo studies have indicated that *M. charantia* exerts an antioxidative effect by enhancing the activity of superoxide dismutase and glutathione peroxidase and by modulating the NF-κB pathway (Table 1 and Table 2) [27,36,43,55]. Moreover, the antioxidative activity of momordicine I reduces oxidative stress in the cardiovascular system, thereby preventing endothelial dysfunction and atherosclerosis [27]. Additional clinical trials are essential to substantiate the antioxidative activity of bioactive compounds in *M. charantia*. Evidence indicates that excessive oxidative stress and inflammation are closely related pathophysiological processes that can activate each other [56]. Chronic inflammation plays a crucial role in the development of various diseases, such as type 2 diabetes, metabolic syndrome, cardiovascular diseases, cancer, and neurodegenerative diseases [57]. Various extracts of *M. charantia* have been found to regulate inflammation mainly through the NF-κB signaling pathway, TNF-α-induced inflammation, MAPK phosphorylation, and decreases in iNOS and IL-1β expression (Table 1 and Table 2) [12,34,38,58,59,60]. These findings indicate that *M. charantia* extracts exert an anti-inflammatory effect by targeting several key inflammatory pathways, thereby offering protection against cardiovascular damage [9]. Hyperlipidemia is a potential risk factor for cardiovascular diseases. *M. charantia* juice can act as a hypolipidemic agent, reducing serum total cholesterol, low-density lipoprotein cholesterol, and triglycerides, with effects comparable to those observed in Norwegian rats treated with a statin drug [61]. Other in vivo studies have demonstrated that the *M. charantia* extract reduced lipid peroxidation in the adipose tissue and the blood lipid level in rats [40,41]. An in silico analysis revealed that momordicine I reduces lipids through mechanisms involving lipophagy [52]. Hypertension is another risk factor for cardiovascular diseases. The methanol extract of *M. charantia* exerted an antihypertensive effect on Dahl salt-sensitive rats [20]. In addition, the ethyl acetate fraction from the 80% ethanolic extract of *M. charantia* leaves exhibited the highest inhibition activity against angiotensin-converting enzyme [62]. In terms of myocardial health, momordicine I was found to inhibit isoproterenol-induced cardiomyocyte hypertrophy and diabetes-associated cardiac fibrosis [8,28]. Furthermore, emerging evidence suggests a link between gut microbiota composition and cardiovascular health. *M. charantia*, containing momordicine I, modulates gut microbiota composition and increases SCFA production, thus inhibiting cardiac fibrosis [24,63,64,65]. Finally, CAD is the most common type of cardiovascular illness. Momordicine I exhibits therapeutic potential for CAD through multiple mechanisms, including lipid reduction and anti-inflammatory actions. An in vivo study demonstrated that dietary *M. charantia* could attenuate the development of atherosclerosis in ApoeE^−/−^ mice by reducing triglycerides and inflammation [66]. In addition, bioactive compounds in *M. charantia* exerted an anti-inflammatory effect by inhibiting the NF-κB-NLR family pyrin domain-containing protein 3 (NLRP3) pathway in RAW 264.7 macrophages [67]. The NLRP3 inflammasome is a crucial risk factor for vascular inflammation and atherosclerosis [68]. An in vivo study reported the beneficial effects of the *M. charantia* extract on vascular complications in diabetic rats [41]. Furthermore, cucurbitane-type triterpenoids could inhibit the proliferation of rat aortic vascular smooth muscle cells [37].

**Figure 3:**
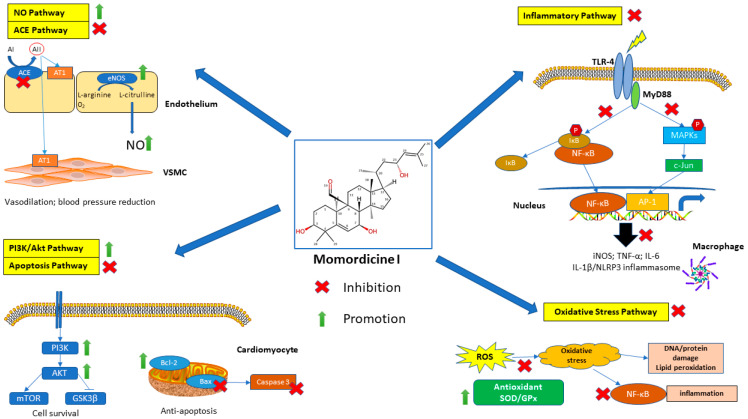
Mechanistic pathways of momordicine I in cardiovascular health. Proposed mechanistic pathways through which momordicine I exerts its cardiovascular benefits. Momordicine I influences several key pathways: Nitric Oxide (NO) Pathway: Upregulates endothelial nitric oxide synthase (eNOS), enhancing NO production and vasodilation, thus reducing blood pressure. Angiotensin-Converting Enzyme (ACE) Pathway: Inhibits ACE activity, reducing angiotensin II levels and vasoconstriction. PI3K/Akt Pathway: Activates the PI3K/Akt signaling pathway, promoting cardiomyocyte survival and protecting against ischemic injury. Oxidative Stress Pathway: Reduces reactive oxygen species levels and boosts antioxidant enzyme activity, mitigating oxidative damage. Inflammatory Pathway: Reduces the expression of proinflammatory cytokines, such as TNF-α and IL-6, reducing inflammation and preventing atherosclerosis. Apoptosis Pathway: Inhibits apoptotic signaling by modulating the Bax/Bcl-2 ratio, reducing cardiomyocyte apoptosis and protecting against myocardial injury. These pathways collectively contribute to the cardiovascular protective effects of momordicine I, highlighting its therapeutic potential in treating cardiovascular diseases.

**Figure 4:**
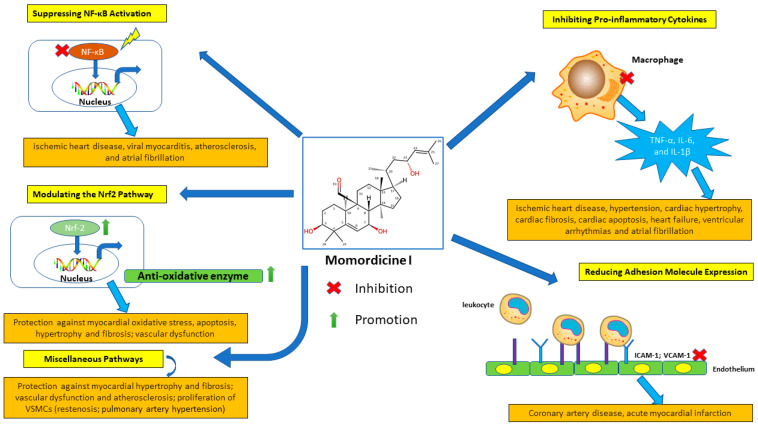
Anti-inflammatory effects of momordicine I in cardiovascular disease models. Illustration of the anti-inflammatory effects of momordicine I in cardiovascular disease models. Momordicine I reduces inflammation through the following mechanisms: Inhibiting Pro-inflammatory Cytokines: Reduces the levels of TNF-α, IL-6, and IL-1β, which are key mediators in the inflammatory response associated with cardiovascular diseases. Reducing Adhesion Molecule Expression: Lowers the expression of adhesion molecules, such as ICAM-1 and VCAM-1, thereby reducing the adhesion and infiltration of inflammatory cells into the vascular endothelium. Suppressing NF-κB Activation: Inhibits the activation of NF-κB, a transcription factor that plays a central role in the inflammatory process, leading to decreased transcription of inflammatory genes. Modulating the Nrf2 Pathway: Activates the Nrf2 pathway, enhancing the expression of antioxidant proteins that protect against inflammatory damage. Miscellaneous Pathways: Suppresses PLA2G6 and DGK-ζ, inhibits diabetes-associated cardiac fibrosis by increasing SCFAs and the TGF-β1/Smad pathway, and downregulates the c-Met/STAT3 pathway (Table 2). These mechanisms demonstrate the potential beneficial effects of momordicine I, indicating its potential as an adjuvant in cardiovascular disease therapy.

Momordicine I can downregulate the c-Met/STAT3 signaling pathway [14]. STAT3 is a transcription factor that mediates intracellular signal transduction involved in vascular smooth muscle cell proliferation [69]. In summary, momordicine I is a promising therapeutic agent for managing cardiovascular diseases due to its diverse effects on glucose metabolism, cardiac remodeling, oxidative stress, gut microbiota modulation, and inflammation. Additional studies should be conducted to fully elucidate the protective roles of momordicine I in cardiovascular health. The proposed mechanisms underlying the cardiovascular benefits of momordicine I are summarized in Figure 3 and Figure 4.

## 4. Safety and Toxicology

### 4.1. Toxicological Profile

Assessing the toxicological profile of momordicine I is crucial for determining its safety and potential risks. The literature on *M. charantia*, the source of momordicine I, generally indicates a favorable safety profile. A systematic review and meta-analysis by Chattopadhyay et al. demonstrated the effectiveness and safety of Ayurvedic medicines, including *M. charantia*, in managing type 2 diabetes mellitus [70]. Similarly, Çiçek (2022) reported the diabetes-related bioactivities of *M. charantia*, indicating the importance of quality control and its safety when used appropriately [71]. However, some studies have identified potential adverse effects. For example, Du et al. (2021) reported cardiotoxicity associated with *Cochinchina momordica* seed extract, demonstrating the need for cautious use and further investigations into its safety profile [72]. These findings suggest that although *M. charantia* and its bioactive compound, momordicine I, have substantial therapeutic potential, particularly in diabetes management, the risk of adverse effects, such as cardiovascular toxicity, exists. This risk necessitates comprehensive safety assessments and monitoring, particularly for high-risk populations or during prolonged use. Therefore, further research, including rigorous preclinical and clinical studies, is essential to fully determine the safety profile of momordicine I.

### 4.2. Side Effects

Momordicine I, derived from *M. charantia*, demonstrates substantial therapeutic potential, but concerns regarding its potential side effects remain. Some reported adverse events associated with *M. charantia* use are gastrointestinal symptoms, such as nausea, anorexia, abdominal discomfort, and soreness; foamy urine; and skin rashes [73]. The consumption of traditional medicinal plants containing *M. charantia* during pregnancy is particularly concerning. Bernstein et al. (2021) highlighted the potential risk of *M. charantia* to maternal and fetal health and advised caution [74]. Adarmanabadi et al. (2024) investigated the pharmacotherapeutic potential of *M. charantia*, particularly for treating age-related neurological diseases, and indicated the importance of considering potential side effects, especially in vulnerable populations [75]. They recommend conducting additional in vitro and in vivo studies to fully understand its mechanisms and clinical trials to evaluate its safety in patients [75]. Chung et al. (2022) examined the acute and subchronic toxicity of *M. charantia* seed extract in Wistar rats and highlighted the necessity of comprehensive safety assessments [76]. In addition, Doğaroğlu et al. (2024) found that *M. charantia* extract-based nanoparticles exhibit antibacterial properties, which could also pose toxicity concerns that warrant further investigation [77]. Zafar et al. (2023) evaluated the antioxidant potential of medicinal plants, including *M. charantia* [55]. They reported that although antioxidants offer numerous health benefits, excessive consumption may lead to adverse effects, highlighting the importance of moderation. Moreover, Ali et al. (2022) investigated the effects of *M. charantia* on insulin-immunoreactive pancreatic beta cells and blood glucose levels in diabetic rats. They reported promising outcomes for glucose regulation but also indicated the need to monitor for adverse reactions [78]. Thus, careful monitoring and additional research are critical to fully understand the potential side effects of momordicine I derived from *M. charantia*, ensuring its safe and effective use in various therapeutic contexts.

### 4.3. Contraindications and Drug Interactions

Interactions between *M. charantia* and chemotherapeutic agents have been reported in the literature. Unsal et al. (2022) documented a case of acute pancreatitis resulting from an interaction between *M. charantia* and pazopanib, a chemotherapeutic agent [79]. This finding highlights the potential risks associated with combining *M. charantia* with certain tyrosine kinase inhibitors, such as pazopanib. In addition, computational studies have explored potential drug interactions. Adelusi et al. (2021) investigated the inhibitory potential of bioactive compounds present in *M. charantia* against the Keap1-Kelch protein and suggested that these compounds can interact with certain medications [80]. Furthermore, synergistic effects with chemotherapy drugs have been examined. Chan et al. (2020) demonstrated that the MAP30 protein from *M. charantia* exhibited synergistic activity with cisplatin against ovarian cancer, indicating potential interactions between *M. charantia* and chemotherapeutic agents [81]. In terms of neuroprotection, Huang et al. (2018) reported that although *M. charantia* enhances neuroprotection, caution is advised when administering it with lithium chloride for the treatment of Alzheimer’s disease due to potential side effects [82]. Furthermore, Kuok et al. (2017) demonstrated that herbal extracts, including *M. charantia*, exerted synergistic antibacterial effects in combination with antibiotics against methicillin-resistant *Staphylococcus aureus*, suggesting their potential interactions with antibiotics [83]. Thus, on the basis of these findings, potential contraindications and drug interactions should be carefully considered to ensure the safe and effective use of *M. charantia* in various therapeutic contexts.

## 5. Conclusions

### 5.1. Summary of Key Findings

In summary, preclinical studies have indicated that momordicine I exhibits substantial cardiovascular effects, including antihypertensive, anti-inflammatory, antihypertrophic, antifibrotic, and antioxidative properties. These findings indicate its potential as a therapeutic agent for cardiovascular diseases. However, the careful consideration of potential side effects and contraindications, especially in individuals with pre-existing cardiovascular conditions, is necessary. In addition, caution is warranted regarding potential drug interactions. Additional research, including clinical trials, is warranted to validate these findings and evaluate the translational potential of momordicine I in clinical practice.

### 5.2. Future Research and Clinical Directions

Although preclinical studies have provided valuable insights into the cardiovascular effects of momordicine I, several research gaps remain. Future studies should focus on elucidating the precise mechanisms underlying the cardiovascular effects of momordicine I, including its interactions with specific molecular targets and signaling pathways. In addition, clinical trials are needed to validate the efficacy and safety of momordicine I in humans, particularly in those with cardiovascular diseases. Comparative studies with existing cardiovascular medications can help determine whether momordicine I could serve as an adjunct or alternative therapy. Furthermore, the long-term effects of momordicine I on cardiovascular outcomes and overall health should be investigated. In addition, exploring the pharmacokinetics of momordicine I, including its ADME in humans, can provide valuable insights into its therapeutic potential and help inform dosage regimens. Addressing these research gaps will enhance our understanding of momordicine I and its clinical utility in managing cardiovascular diseases.

### 5.3. Clinical Implications

If further validated, momordicine I holds substantial potential for clinical practice in cardiology. The antihypertensive, anti-inflammatory, antihypertrophic, antifibrotic, and antioxidative effects of momordicine I suggest that it could serve as a valuable adjunct or alternative therapy for managing cardiovascular diseases. Furthermore, the unique mechanisms of action of momordicine I offer the possibility of addressing cardiovascular conditions from novel angles, potentially complementing existing treatment approaches. However, translating momordicine I into clinical practice requires rigorous validation through well-designed clinical trials to establish its efficacy, safety, and optimal dosage regimens. Despite the need for further research, the therapeutic promise of momordicine I highlights its potential to contribute to the range of cardiovascular therapies, offering hope for improved patient outcomes in cardiology.

## Figures and Tables

**Table 1:** Overview of the cardiovascular benefits of bioactive compounds in *Momordica charantia*, summarizing findings from published in vitro, in vivo, in silico, and clinical studies.

Title of the Study	Aim/Methods	Summation of Findings	Compounds or Materials Tested	References
**In vitro studies**				
Suppressive effects of wild bitter gourd (*Momordica charantia* Linn. var. *abbreviate* ser.) fruit extracts on inflammatory responses in RAW264.7 macrophages	To examine the anti-inflammatory effect of *M. charantia* on lipopolysaccharide (LPS)-stimulated RAW264.7 macrophages.	The ethanol extract of *M. charantia* reduced LPS-induced inflammatory responses by modulating NF-κB activation.	*M. charantia* extract	[34]
Transport in Caco-2 Cell Monolayers of Antidiabetic Cucurbitane Triterpenoids from *Momordica charantia* Fruits	To investigate the gastrointestinal transport of a triterpenoid-enriched n-butanol extract of *M. charantia* by using a Caco-2 monolayer system	The findings demonstrated the transport of cucurbitane triterpenoids in human intestinal epithelial cell monolayers.	cucurbitane triterpenoids	[15]
In vitro and in vivo α-amylase and α-glucosidase inhibiting activities of the protein extracts from two varieties of bitter gourd (*Momordica charantia* L.)	To examine the inhibitory effect of protein extracts from two varieties of bitter gourd	Protein extracts from two varieties of bitter gourd inhibited α-amylase and α-glucosidase In vitro	*M. charantia* extract	[35]
Antioxidant activity of various extracts of selected gourd vegetables	To evaluate the antioxidative activity of methanolic, ethanolic, and butanolic extracts of selected gourd vegetables.	Extracts of *M. charantia* revealed significantly higher (*p* < 0.05) antioxidative activity than did the extracts of other remaining vegetables.	*M. charantia* extract	[36]
Inhibition of Proliferation of Vascular Smooth Muscle Cells by Cucurbitanes from *Momordica charantia*	To determine the effects of cucurbitane-type triterpenoids from the fruits of *M. charantia* on vascular smooth muscle cells	The triterpenoids inhibited the proliferation of vascular smooth muscle cells.	cucurbitane triterpenoids	[37]
Inhibitory Effects of Momordicine I on High-Glucose-Induced Cell Proliferation and Collagen Synthesis in Rat Cardiac Fibroblasts	To evaluate the effects of momordicine I (0.3 and 1 μM) pretreatment on rat cardiac fibroblasts cultured in a high-glucose (25 mM) medium	The antifibrotic effect of momordicine I was mediated, at least partially, by the inhibition of the TGF-β1/Smad pathway, reducing fibroblast proliferation and collagen synthesis through Nrf2 activation.	Momordicine I	[8]
*Momordica charantia* Inhibits Inflammatory Responses in Murine Macrophages via Suppression of TAK1	To investigate the anti-inflammatory effect of *M. charantia* on LPS-stimulated RAW264.7 macrophages.	The methanol extract of *M. charantia* exerted an anti-inflammatory activity by reducing the action of transforming growth factor β-activated kinase 1, which also affected the activation of NF-κB and AP-1.	*M. charantia* extract	[38]
Momordicine-I, a Bitter Melon Bioactive Metabolite, Displays Anti-Tumor Activity in Head and Neck Cancer Involving c-Met and Downstream Signaling.	To identify momordicine I and evaluate its role in a head and neck cancer (HNC) preclinical mouse model.	Momordicine I inhibited HNC cell growth and c-Met/STAT3 signaling. However, momordicine I had a minimal effect on human normal oral keratinocytes.	Momordicine I	[14]
Cytotoxic and Anti-Inflammatory Triterpenoids in the Vines and Leaves of *Momordica charantia*	To analyze the cytotoxic and anti-inflammatory effects of cucurbitane-type triterpenoid species and the mechanisms underlying these effects.	Momordicine I exerted deleterious effects on cell lines at concentrations greater than 10 or 20 µM. The momordicine I isomer TCD exhibited anti-inflammatory activity in LPS-stimulated RAW 264.7 cells by inhibiting the NF-κB pathway and enhancing the expression of Nrf2/HO-1.	Momordicine I	[9]
Momordicine I alleviates isoproterenol-induced cardiomyocyte hypertrophy through suppression of PLA2G6 and DGK-ζ	To evaluate the effect of momordicine I, a triterpenoid compound extracted from *M. charantia* L., on isoproterenol (ISO)-induced hypertrophy in rat H9c2 cardiomyocytes. This study used 12.5 μg/mL of momordicine I.	Momordicine I inhibited ISO-induced upregulation of mRNA levels and protein expression of PLA2G6 and DGK-ζ. Collectively, it alleviated ISO-induced cardiomyocyte hypertrophy.	Momordicine I	[28]
**In vivo studies**				
Effect of bitter gourd (*Momordica charantia*) on glycaemic status in rats with streptozotocin-induced diabetes.	To evaluate the effects of bitter gourd powder, incorporated at a 10% level in place of an equivalent amount of corn starch in the AIN-76 basal diet, over a period of 45 days	Improved diabetic status, evidenced by a significant reduction in the glomerular filtration rate	*M. charantia*	[39]
Antidiabetic effects of bitter gourd extracts in insulin-resistant db/db mice	To determine the effects of the whole fruit powder, a lipid fraction, a saponin fraction, or the hydrophilic residue of bitter gourd administered at a daily dosage of 150 mg/kg body weight for 5 weeks	Reduction in glycated Hb levels in all treatment groups. Specifically, the groups treated with saponin and lipid fraction showed decreases in lipid peroxidation in the adipose tissue and protein tyrosine phosphate 1 B activity in skeletal muscles.	*M. charantia* extract	[40]
Effect of *Momordica charantia* fruit extract on vascular complication in type 1 diabetic rats	To investigate the effects of a fruit extract administered at a rate of 1.5 g/kg of rats for 28 days after induction of diabetes	Improvement in vascular function, evidenced by decreased blood pressure, lipid levels, aortic tissue MDA levels, and increased aortic nitrous oxide levels.	*M. charantia* extract	[41]
In vitro and in vivo α-amylase andα-glucosidase inhibiting activities of the protein extracts from two varieties of bitter gourd (*Momordica charantia* L.)	To determine the effects of protein extracts derived from bitter gourd cultivars and fed to rats at a dosage of 10 mg/kg body weight. Blood samples were drawn after 10, 30, 60, and 120 min of oral administration.	Significant reduction in peak blood glucose levels.	*M. charantia* extract	[35]
Hypoglycemic and hypolipidemic effects of *Lactobacillus fermentum*, fruit extracts of *Syzygium cumini* and *Momordica charantia* on diabetes induced mice.	To investigate the effects of the aqueous and ethanol extracts of bitter gourd administered at a rate of 200 mg/kg weight of mice for 3 weeks	Significant reduction in blood glucose levels.	*M. charantia* extract	[42]
*Momordica charantia* polysaccharides ameliorate oxidative stress, hyperlipidemia, inflammation, and apoptosis during myocardial infarction by inhibiting the NF-κB signaling pathway	To evaluate the effect of the *M. charantia* extract on endothelial dysfunction in myocardial infarction.	Pretreatment with *M. charantia* polysaccharides (150 or 300 mg/kg) for 25 days significantly inhibited increases in heart weight, the heart-weight-to-body-weight ratio, and infarction size. This myocardial protective effect is potentially due to the enhancement of the antioxidant defense system through NF-κB pathways and anti-apoptosis through regulation of Bax, caspase-3, and Bcl-2.	*M. charantia* extract	[43]
Minerals and phytochemical analysis of bitter melon fruits and its components in some indigenous and exotic cultivars.	To investigate the effects of administering skin, flesh, and fruit powder from bitter melon at doses of 150 and 300 mg/kg body weight for 56 days	A decrease in the blood glucose level and an increase in the serum insulin level at the dosage of 300 mg.	*M. charantia*	[44]
A triterpenoid-enriched extract of bitter melon leaves alleviates hepatic fibrosis by inhibiting inflammatory responses in carbon tetrachloridetreated (CCl4) mice	To assess the efficacy of a triterpenoid-enriched extract administered at 100 or 150 mg/kg daily via oral gavage, starting one week before and continuing through CCl4 administration	Amelioration of hepatic fibrosis by regulating inflammatory cytokine secretion and α-smooth muscle actin expression in the liver, reducing collagen accumulation.	cucurbitane triterpenoids	[45]
Momordicine-I, a Bitter Melon Bioactive Metabolite, Displays Anti-Tumor Activity in Head and Neck Cancer Involving c-Met and Downstream Signaling.	To identify momordicine I and evaluate its role in head and neck cancer preclinical mouse model.	The Cmax values were 18 µM and 0.5 µM after the single 20 mg/kg IP and PO dose, respectively. No adverse events were observed in the IP dosing group. A significant reduction in the expression of c-Met and its downstream molecule c-Myc was observed in the momordicine I- treated group compared with the untreated group.	Momordicine I	[14]
Cytotoxic and Anti-Inflammatory Triterpenoids in the Vines and Leaves of *Momordica charantia*	To analyze the anti-inflammatory effects of cucurbitane-type triterpenoid species	The momordicine I isomer TCD exhibited anti-inflammatory activity. TCD ameliorated ear edema, a sign of ear inflammation, in the mouse model.	Momordicine I	[9]
*Momordica charantia* Extract Confers Protection Against Hypertension in Dahl Salt-Sensitive Rats	To determine the antihypertensive effects of *M. charantia* water extracts	Alleviation of oxidative stress and salt-induced hypertension in Dahl/SS rats	*M. charantia* extract	[20]
**Clinical studies**				
Hypoglycemic effect of bitter melon compared with metformin in newly diagnosed type 2 diabetes patients	To assess the effect of bitter melon capsules containing 500 mg of dried fruit pulp with 0.04–0.05 (*w*/*w*) of charantin, administered at doses of 500/1000/2000 mg per day and that of with 1000 mg of metformin per day for 4 weeks	Modest hypoglycemic effects were observed, but they were less substantial than those achieved with 100 mg of metformin per day	*M. charantia*	[46]
Wild bitter gourd improves metabolic syndrome: a preliminary dietary supplementation trial	To evaluate the effects of supplementing 42 eligible participants (21 men and 21 women) with a mean age of 45.7 ± 11.4 years (23 to 63 years) with 4.8 g of lyophilized bitter melon powder in capsules daily for 3 months	The incidence rate of metabolic syndrome decreased when compared with baseline. The waist circumference also significantly decreased.	*M. charantia*	[47]
“Pilot study: hypoglycemic and antiglycation activities of bitter melon (*Momordica charantia* L.) in type 2 diabetic patients	To determine the effects of continuous intake of 6 g/day of *M. charantia* L. dried-fruit pulp compared with placebo for 16 weeks.	Significant declines in the levels of total advanced glycation end-products in serum after the intervention	*M. charantia*	[48]
Evaluation of supplementation of Bitter gourd fermented beverage to diabetic subjects.	To investigate the effect of a 45 mL daily morning drink of bitter gourd fermented beverage	Significant reductions in the symptoms of diabetes and fasting and post prandial blood sugar levels were observed.	*M. charantia*	[49]
Preliminary clinical trials of karela, *Momordica charantia*, on non-insulin-dependent diabetes mellitus patients.	To test the effect of powdered bitter gourd made into a tablet containing a 20 mg polypeptide, with a dosage of 4 to 6 tablets per day taken half an hour before meals for 8 weeks.	Effective oral adjunct hypoglycemic effect observed with no reportable clinical side effects	*M. charantia*	[50]
Bitter gourd reduces elevated fasting plasma glucose levels in an intervention study among prediabetics in Tanzania	To explore the effects of daily consumption of 2.5 g of bitter gourd powder over a course of 8 weeks, employing a crossover design with an 8-week study period followed by a 4-week washout.	Lowered fasting plasma glucose levels were noted.	*M. charantia*	[51]
**In silico study**				
Network Pharmacology and Experimental Study of Momordicine I and Momordicine II from Bitter Melon Saponins in Inhibiting Fat Accumulation	To screen for potential ant-obesity compounds in the bitter melon extract through LC/Q-TOF-MS/MS and network pharmacology and to estimate the lipid-lowering effects of these compounds in vivo based on the Kyoto Encyclopedia of Genes and Genomes pathway enrichment analysis	Triterpenoids in the extract could phosphorylate AMPK/mTOR and subsequently promote GLUT4 translocation to the cell membrane, thereby eliminating hyperglycemia both in vivo and in vitro. Momordicine I was identified as the core component likely responsible for treating obesity according to the compound-target-disease-pathway network. It exerted its lipid reduction capacity through daf-16/FoxO1 and hlh-30/TFEB-mediated lipophagy, consistent with the predicted AMPK/mTOR signaling pathway.	Momordicine I	[52]

**Table 2:** Potential biochemical and molecular pathways modulated by momordicine I.

Pathway	Description	Key Findings	References
Glucose metabolism	Modulation of glucose metabolism pathways.	Inhibits α-amylase and α-glucosidase; improves insulin sensitivity; stimulates GLUT-4 translocation; activates the AMPK signaling pathway; increases the expression of PPARγ; and acts as a GLP-1 secretagogue	[7,23,30,31,35]
Lipid metabolism	Regulation of lipid metabolism pathways.	Reduces lipid accumulation by inhibiting lipogenic enzymes and lipid peroxidation and increases lipophagy	[40,41,52]
Inflammatory	Inhibition of inflammatory mediators and pathways.	Inhibits the NF-κB pathway, reduces TNF-α-induced inflammation, inhibits MAPK phosphorylation, and reduces iNOS and IL-1β/NLRP3 inflammasome expression	[12,34,38,58,59,60]
Oxidative stress	Reduction of oxidative stress through the modulation of antioxidant enzymes.	Enhances antioxidant defenses by increasing the activity of superoxide dismutase and glutathione peroxidase or through the NF-κB pathway	[27,36,43,55]
Apoptosis	Modulation of apoptosis-related proteins and pathways.	Promotes anti-apoptosis by downregulating Bax/caspase-3 and upregulating Bcl-2 protein expression	[43]
Cardiovascular diseases	Protection against cardiovascular-related disorders.	Alleviates cardiomyocyte hypertrophy by suppressing PLA2G6 and DGK-ζ; exerts an antihypertensive effect by inhibiting ACE; inhibits diabetes-associated cardiac fibrosis by increasing SCFA production, activating Nrf2 or inhibiting the TGF-β1/Smad pathway; suppresses the NF-κB-NLRP3 pathway; and downregulates the c-Met/STAT3 pathway	[8,14,24,28,37,62,63,67]

## Data Availability

Not applicable.

## Abbreviations

ADME	absorption: distribution: metabolism, and excretion
AMPK	AMP-activated protein kinase
CAD	coronary artery disease
*c* *-* *Met*	c-mesenchymal–epithelial transition factor
DGK-ζ	diacylglycerol kinase-ζ
ERK	extracellular signal-related kinase
GLP-1	glucagon-like peptide 1
GLUT4	stimulation of glucose transporter type 4
GPx	glutathione peroxidase
IKK	*inhibitor kappa B kinase*
IL-1β	interleukin-1β
IL-6	interleukin-6
iNOS	inducible nitric oxide synthase
LPS	lipopolysaccharide
MAPKs	mitogen-activated protein kinases
NF-κB	nuclear factor kappa-light-chain enhancer of activated B cells
Nrf2/HO-1	nuclear factor erythroid 2-related factor 2/heme oxygenase-1
PLA2G6	phospholipase A2 group VI
PPARγ	peroxisome proliferator-activated receptor gamma
SCFAs	short-chain fatty acids
Smad2/3	suppressor of mothers against decapentaplegic 2/3
SOD	superoxide dismutase
STAT3	signal transducer and activator of transcription 3
TCM	Traditional Chinese Medicine
*TGF*-*β1*	transforming growth factor-*β1*
TNF-α	tumor necrosis factor-α
